# Kiosk versus In-person Screening for Alcohol and Drug Use in the Emergency Department: Patient Preferences and Disclosure

**DOI:** 10.5811/westjem.2015.1.24121

**Published:** 2015-03-10

**Authors:** Abigail Hankin, Leon Haley, Amy Baugher, Kia Colbert, Debra Houry

**Affiliations:** * Emory University, Department of Emergency Medicine, Atlanta, Georgia; † Grady Health System, Atlanta, Georgia

## Abstract

**Introduction:**

Annually eight million emergency department (ED) visits are attributable to alcohol use. Screening ED patients for at-risk alcohol and substance use is an integral component of screening, brief intervention, and referral to treatment programs, shown to be effective at reducing substance use. The objective is to evaluate ED patients’ acceptance of and willingness to disclose alcohol/substance use via a computer kiosk versus an in-person interview.

**Methods:**

This was a cross-sectional, survey-based study. Eligible participants included those who presented to walk-in triage, were English-speaking, ≥18 years, were clinically stable and able to consent. Patients had the opportunity to access the kiosk in the ED waiting room, and were approached for an in-person survey by a research assistant (9am–5pm weekdays). Both surveys used validated assessment tools to assess drug and alcohol use. Disclosure statistics and preferences were calculated using chi-square tests and McNemar’s test.

**Results:**

A total of 1,207 patients were screened: 229 in person only, 824 by kiosk, and 154 by both in person and kiosk. Single-modality participants were more likely to disclose hazardous drinking (p=0.003) and high-risk drug use (OR=22.3 [12.3–42.2]; p<0.0001) via kiosk. Participants who had participated in screening via both modalities were more likely to reveal high-risk drug use on the kiosk (p=0.003). When asked about screening preferences, 73.6% reported a preference for an in-person survey, which patients rated higher on privacy and comfort.

**Conclusion:**

ED patients were significantly more likely to disclose at-risk alcohol and substance use to a computer kiosk than an interviewer. Paradoxically patients stated a preference for in-person screening, despite reduced disclosure to a human screener.

## INTRODUCTION

### Background

In the United States, emergency department (ED) patients are more likely than the general population to report alcohol and substance abuse.^1^,^2^ Eight million ED visits annually are attributable to alcohol,^3^ leading to increased rates of ED crowding due to alcohol-related diseases and injuries.^4^ Furthermore, ED patients with unmet substance abuse treatment needs were 46% more likely to return to the ED within a year than patients who did not report substance abuse problems.^5^

Consistent with Healthy People 2020 recommendations,^6^ the American College of Surgeons Committee on Trauma requires that all Level I trauma centers have a mechanism to identify and provide intervention for trauma patients with at-risk alcohol use.^7^ Screening, brief intervention, and referral to treatment (SBIRT), provides a validated program for alcohol/drug use screening and intervention among ED patients to assess risk for alcohol and substance abuse, reduce future traumas,^3^ and reduce future hazardous drinking.^8^ Positive effects of SBIRT interventions have been shown with respect to multiple health outcomes, including blood pressure, fetal alcohol syndrome, and rates of future ED visits/hospitalizations.^9^ SBIRT tools have also been validated among populations comprised of a variety of racial and ethnic groups.^10^

Nevertheless, ED staff rarely assess for substance and alcohol use.^5^ Clinicians have identified a number of barriers to alcohol and drug use screening in the course of an ED visit, including lack of resources for patients who are identified as at risk or dependent, insufficient education and training in screening for drug and alcohol use, and lack of specific treatment protocols^11^ Computer-based screening tools, such as kiosks, have been proposed to help alleviate the burden on ED staff and reduce social desirability bias that may discourage patients from reporting as alcohol and substance use.^12^ Previous studies have suggested that ED kiosks are accepted by most patients,^2^ are easy to use,^13^ can help patients better recall health promotion advice,^14^ and can effectively screen at-risk drinkers.^15^,^16^ Computer-administered brief substance-abuse interventions have been shown to be effective at reducing hazardous drinking at 12-month follow up.^17^,^18^

In this study, we aimed to 1) compare results of alcohol and drug disorder screening via kiosk vs. in-person modalities, and 2) compare population capture for patients screened by kiosk vs. in-person, estimating the additional yield offered by having kiosk screening available 24 hours a day, 7 days a week. We hypothesized that computerized kiosk screenings for alcohol and drug use would yield similar disclosure rates when compared with in-person screening.

## METHODS

### Study Design

This study consisted of a cross-sectional, survey-based study of patients presenting to the ED from June through September 2013. Patients were screened for alcohol and substance use with the Alcohol Use Disorders Identification Test (AUDIT)^19^ and the Drug and Alcohol Screening Test (DAST-10),^20^ via one or both of the following convenience methods: a) computer-based kiosk general health screening including multiple general physical and mental health surveys, or b) in-person survey administration in a private area of the ED waiting room or a private patient care room (screening method used for each patient was determined based on patient preference, and based on time of day of patient arrival, as the research assistant (RA) was present in the ED 40 hours per week.

### Setting

This study was conducted in a large, inner-city, safety net hospital in the southeastern United States with a Level I trauma center. The annual ED volume exceeds 120,000 patient visits. Patients at this ED are predominantly African American, low socioeconomic status, and uninsured. The institutional review board at the hospital and the affiliated school of medicine approved the study protocol.

### Inclusion/Exclusion Criteria

Participants were included if they were at least 18 years old, presenting to walk-in triage for medical care at the ED, able to understand English, and able to provide consent. We excluded participants if they presented to the ED by ambulance, were younger than 18 years, critically ill or medically unstable, acutely intoxicated, presenting for an acute psychiatric complaint, incarcerated, or otherwise unable to provide informed consent. The kiosk survey was self-initiated and did not require inclusion criteria to complete; however, any patient under the age of 18 was excluded from this analysis.

### Participant Recruitment

The kiosk computer was located in the ED waiting room, and was available for use 24 hours per day, every day. The kiosk screening included multiple validated instruments focusing on general health, mental health, intimate partner violence risk, and alcohol/substance use. For drug and alcohol use screening, the kiosk screening used the AUDIT and the DAST-10. Patients were informed by signs posted in the ED and/or a dedicated project RA that they could use the kiosk to obtain a “free health screening.” Results of the kiosk screening were provided to patients via a computer print-out, with relevant education and support services referrals. Information about patient responses to the kiosk screening was not placed into the medical record or otherwise available to the ED care team.

**Figure 1:**
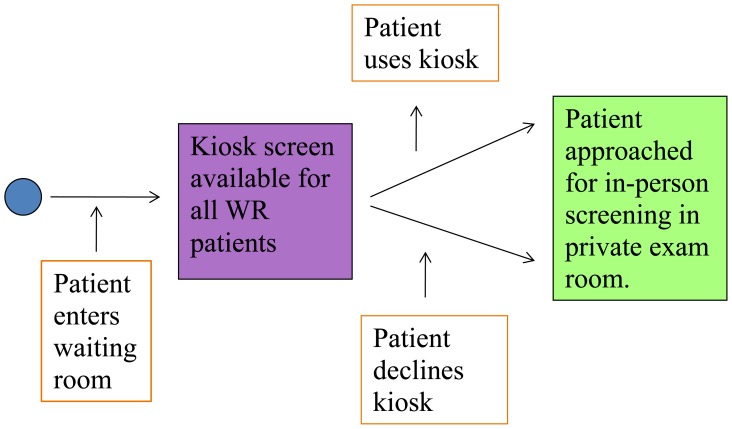
Patient flow diagram of participation in alcohol and drug use screening, using kiosk and in-person modalities. *WR,* waiting room

For participation in the in-person screening, a trained RA was present in the ED Monday through Friday, 9am–5pm. The RA approached eligible patients in both the ED waiting room and in patient care rooms during naturally occurring downtime in the patient’s medical care (such as while awaiting lab or imaging tests to occur or while awaiting test results). Patients were eligible for in-person screening participation regardless of prior completion of the kiosk screening. When approached for in-person screening, participants were informed that the RA was surveying people about health behaviors and survey modality preferences, and that participation was voluntary, confidential, and any choice to agree/decline to participate would have no bearing on the patients’ medical care. All participants provided signed informed consent (Figure 1).

### Survey Instruments

#### Kiosk Screening

The kiosk presented a variety of survey instruments focused on demographics, nutrition, intimate partner violence, sexual health, as well as the AUDIT and DAST-10 for alcohol and drug use screening. Only demographics and alcohol/drug survey items were included in the analysis described here. All kiosk survey responses were automatically collected using a Microsoft Access database. Participants who disclosed any adverse health behaviors were provided print outs with referrals to appropriate local services.

We assessed alcohol use with the AUDIT, a previously validated 10-item survey.^21^ The range of possible AUDIT scores is 0–40. In standard practice, AUDIT scores are translated to alcohol use risk categories: low risk (0–7), moderate risk (8–15), high risk (16–19), or dependent (20 or higher). The AUDIT has been previously validated with an internal consistency of α=0.83^22^ compared to biomarkers, the AUDIT has been shown to have a sensitivity=0.98 and specificity=0.34 at the ≥8 threshold.^21^

Substance use was assessed with the DAST-10,^20^ a previously validated 10-item survey (Appendix) with an internal consistency of α=0.94.^23^ Possible DAST-10 scores range from 0–10. The DAST-10 was categorized thus: lowrisk (0–2) or high-risk (≥3) drug use. Using a threshold of ≥3, the DAST-10 has been shown to have a sensitivity=0.41 and specificity=0.99 relative to biomarkers.^23^

#### In-person survey

The in-person survey items included patient demographics, alcohol use, drug use, and screening modality preferences. Demographic data collected included race, gender, age, employment, education, and marital status. Alcohol use was measured via the AUDIT and the DAST-10. After completion of the other survey instruments, patients were asked a brief series of questions regarding attitudes about and comfort with alcohol and drug screening via computer-based vs. in-person screening. The preferences instrument was modified from a pre-existing survey of patient preferences regarding screening modality of choice for patients surveyed about intimate partner violence.^24^ This survey was not included in the kiosk screening.

Participants were considered “at risk” if they scored ≥8 on the AUDIT or ≥3 on the DAST-10, based on previously established standards for SBIRT interventions.^25^,^26^ Participants whose screening results showed that they were “at risk” for either alcohol or drug abuse during the in-person survey were offered immediate referral to a trained substance abuse specialist.

All dual participants took the kiosk survey prior to the in-person survey, due to both study and clinical patient flow requirements.

### Data Analysis Methods

We summarized and compared demographic characteristics by kiosk status (kiosk only, in-person only, or dual) using a chi-square test to assess for independence between kiosk status and categorical demographic variables and analysis of variance (ANOVA) to assess for independence between kiosk status and age. We assessed differences among participant screening modality preferences using a chi-square test.

Among patients who used only one screening modality, alcohol and modality were compared using a Cochran Mantel-Haenszel chi-square test; drug use and modality were compared using a chi-square test. Among patients screened via both modalities, we compared alcohol and modality using a Friedman’s chi-square test for repeated measures; drug use and modality were compared using McNemar’s test.

To account for missing data, we conducted an intention-to-treat analysis comparing alcohol/drug use to screening modality. All tests described in the previous paragraph were conducted under the conservative assumption that any missing patient was categorized in the lowest possible alcohol/drug risk category.

To address the second aim of population capture by time of day, we summarized when participants took each screening modality, and quantified the additional yield by having the kiosk available during off-hours. Business hours were categorized as Monday through Friday, 9am–5pm off-hours were defined as any other time of day or week. We also summarized which time of day at-risk participants were more likely to take the kiosk. All data were analyzed using SAS Version 9.3 (SAS Institute, Cary, NC).

## RESULTS

Among the 569 participants approached for in-person screening, 382 consented (67.1%). Among patients who did not consent, reasons for refusal included not feeling well (n=66), not interested (n=80), wariness of the study (n=13), lack of English proficiency (n=13), busy (n=9), and other (n=4). During the same period of time, 978 total patients initiated the kiosk screening, with 154 patients participating in both kiosk and in-person screening. Among the remaining 824 participants who only attempted the kiosk, 620 (75.2%) completed any portion of the AUDIT or DAST-10 instruments.

Participants who took the kiosk were significantly less likely than participants who took the in-person survey to complete both the AUDIT and DAST-10 instruments (16.2% vs. 99.9%, p<0.0001). Among participants who only took the in-person modality, 229 participants completed the AUDIT (100%) and 228 completed the DAST-10 (99.6%). Among the 620 participants who attempted only the kiosk, 461 completed the AUDIT (55.9%) and 127 completed the DAST-10 (20.5%). Among the 154 dual participants, 154 completed the AUDIT in-person (100%), 148 (96.1%) completed the kiosk AUDIT survey, 154 (100%) completed the DAST-10 in person, and 32 completed the DAST-10 on the kiosk (20.8%)

Table 1 summarizes the demographics of participants in each of the three screening categories (kiosk, in-person, dual). Among participants who took only the in-person screening, 50.2% were male, compared to the kiosk-only and dual participants, who were 59.5% and 42.9% male, respectively. The majority of participants in each screening group were African American, reflecting the overall ED population; however, participants who used the kiosk-only modality were more likely to identify as white, Asian, Hispanic/Latino, or multiracial than the other screening categories. Participants who took only the in-person survey were less likely to have graduated from high school (74.6%) than participants who took only the kiosk (83.8%) or participants who took both surveys (90.8%; p*=*0.0001*).*

**Figure 2:**
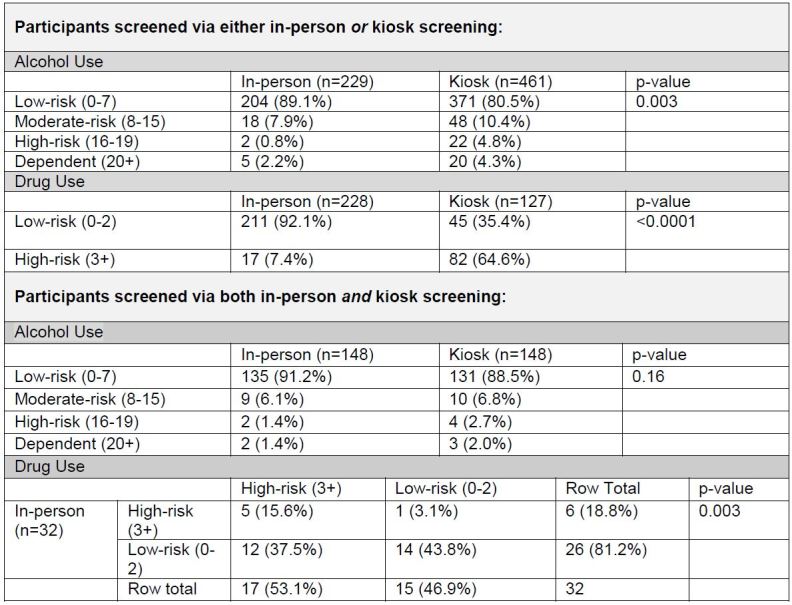
Relationship between reported alcohol/drug use and survey type among participants using only one survey modality and patients screened via both in-person and kiosk screening.

Comparing participants who completed a screening via only one survey modality, either the kiosk or the in-person screening, participants were more likely to report moderate-risk, high-risk, and dependent alcohol use when screened via the kiosk than when screened in-person (p=0.003). This finding extended to the DAST-10 instrument, with participants showing a higher likelihood of reporting high-risk drug use when screened via the kiosk than in-person screening (OR=22.3, 95% CL [12.3–42.2]; p<0.0001) (Figure 2).

Among study participants who were screened via both the kiosk and in-person (Figure 2), there was no difference in disclosure rates for at-risk alcohol use. (p=0.16); however, the participants were more likely to disclose high-risk drug use via the kiosk (p=0.003).

**Figure 3:**
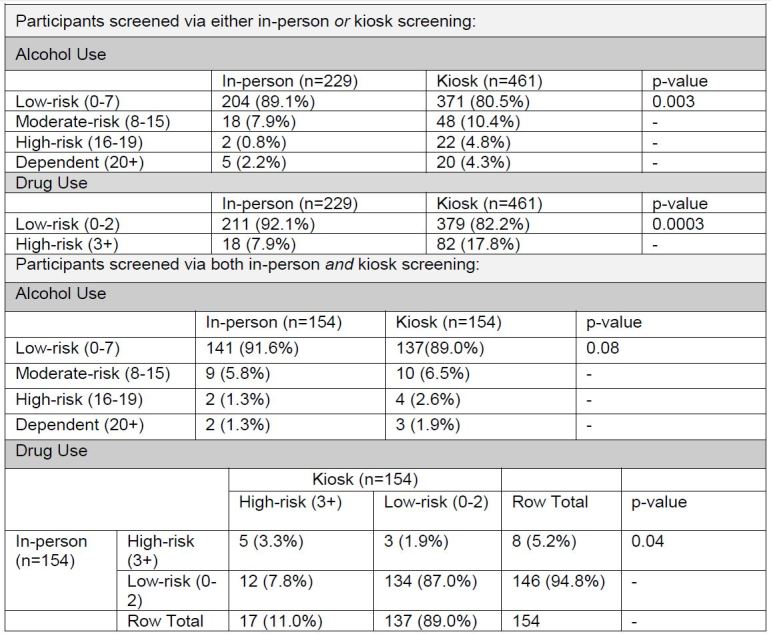
Intention-to-treat analysis of relationship between reported alcohol/drug use and survey type.

Due to the high rate of DAST-10 survey non-completion among kiosk users, we analyzed study results using an intention-to-treat analysis, categorizing all participants who did not complete a survey as low risk. In this analysis, the probability of disclosing high-risk drug use via the kiosk remained significantly higher than via in-person screening (p<0.05), and probability of disclosing high-risk alcohol use remained significant among participants screened via one modality (Figure 3).

When asked about survey modality preferences, participants were more likely to identify in-person screening as “private” (43.6% vs. 34.4%), more likely to elicit honest responses from patients (48.7% vs. 35.3%), and participants identified in-person screening as a modality that they were “more comfortable” with (56.2% vs. 12.1%), and a majority of participants stated they would prefer to complete a 20-minute survey in person (56.2%) (Table 2).

Additionally, to assess the overall yield of kiosk availability at all hours vs. an in-person screener present during routine business hours, we compared capture of participants during business hours vs. off-hours (Table 3). Among kiosk-only participants (n=824), 56.6% of participants took the kiosk survey during off-hours, capturing 106 more participants than during business hours.

Among participants who were at risk and took an in-person survey (n=36), 47.2% accepted a substance abuse specialist referral, 47.2% declined seeing a substance abuse specialist, and 5.6% noted that they were already in a treatment program.

## DISCUSSION

The ED has been identified as an important site to screen for, identify, and intervene among individuals who are at increased risk of poor health outcomes due to health behaviors or exposure to external risk factors, ranging from smoking to unintended pregnancy risk to intimate partner victimization. Due to the safety net role of the ED in our healthcare system, screening in this setting identifies patients who may not have access to primary care or mental healthcare providers, and thus provides an opportunity to identify groups who are most at risk, including minority patients, patients of low socioeconomic status, and patients who are un- or underinsured.

Within this landscape of increased and broadened screening, understanding differing patient attitudes and behaviors when screening via different modalities – most notably, comparing use of computerized to in-person screening - is critical to maximizing the beneficial impact of screening while minimizing the interruption of ED care. This study sheds light on this question, finding that patients were more likely to disclose drug and alcohol use when interacting with a computer kiosk, and that the magnitude of difference was significantly more pronounced when comparing drug screening disclosure with alcohol use disclosure. This finding suggests that patients may be more likely to disclose socially ‘undesirable’ behaviors or behaviors that patients may feel embarrassed about or ashamed of.

On the other hand, patients were less likely to complete the screening instruments on a kiosk terminal when compared with screening by a RA in person, and when asked about their screening preferences, they were more likely to state a preference for in-person screening.

The discrepancy between patients’ stated preference for in-person screening vs. the increased rate of disclosure when being screened by kiosk may be related to relatively lower levels of comfort with technology among patients served by urban, safety-net EDs. This could be addressed by collaboration with consumer groups to create user-friendly, low-literacy-oriented kiosk interfaces and providing brochures or videos to educate patients about the kiosk use and data safety/confidentiality.

It may also be that the kiosk provides a protected, non-judgmental interaction for disclosure of behaviors that a patient might otherwise feel uncomfortable about disclosing, which leads to the increased rate of disclosure of substance/alcohol use. However, patients may state a preference for in-person screening because they may want to have the opportunity to discuss their alcohol/drug use concerns with a health provider. These concerns could be addressed by providing an option for a patient using a kiosk to highlight topics that they want to discuss with their providers.

An additional factor when comparing an in-person screening vs. kiosk screening is screening cost and availability. Within the present study, the RA was present five days per week, from 9am–5pm, whereas the kiosk screening was available at all times. Given the nature of the ED as a healthcare access point that is always open, providing preventive services to patients who present on nights and weekends presents an ongoing challenge. The kiosk screening offers not only round-the-clock availability, but also is not subject to fatigue, a factor that might lead clinicians to skip screening about health behaviors during overnight hours. Providing continuous screening in the ED via the kiosk in our sample allowed us to identify 106 additional at-risk patients than were identified via screening during business hours.

## LIMITATIONS

There are several limitations to this study. Responses were collected through self-report, which is susceptible to recall or social desirability bias. Due to study methodology constraints – specifically the availability of the kiosk screening in the waiting room, while most in-person screenings took place once patients were in an exam room, allowing a private location for in-person screening – all dual-screening participants responded to kiosk questions first, followed by in-person screening. Thus, dual participants may have experienced testing fatigue from answering the same alcohol and drug surveys twice, and this may have biased them towards being less comfortable disclosing sensitive behaviors during the in-person screening.

An additional study limitation was the relatively low numbers of patients screened in person or via both modalities as compared with patients who screened via the kiosk only. This limitation was due in part due to methodological constraints with respect to limited private spaces in which to conduct in-person screening. Furthermore, the fact that patients were allowed to choose a screening modality may have introduced bias. A future, randomized study would address this limitation and would provide further insight into result generalizability. A randomized design would also address the possible bias introduced by the convenience-sample design of this study, as well as potential bias introduced into the current study due to availability of kiosk screening on nights and weekends, when the in-person screening was not available.

Finally, another limitation is the high rate of survey non-completion among kiosk participants. While many participants may have failed to complete the survey due to boredom or because they may have been called for evaluation by the ED nursing staff, it is possible that non-completers were disproportionately likely to screen either positive or negative for substance use. This could be addressed in the future by ensuring that kiosk surveys be kept brief and possibly by enhancing the user interface to increase likelihood of survey completion by participants.

Future studies may examine why participants skip drug use questions on a kiosk, and studies might assess computer-user interfaces that might keep a participant engaged and encourage screening completion in the absence of the social pressure to complete a survey that accompanies an in-person screener. Additionally, given the discrepancy between higher rates of risky drug use disclosure when screening via kiosk vs. participants’ stated preferences for in-person screening, future research into factors that increase acceptability of and confidence in kiosk screening for the general ED population might increase comfort with this screening modality. Finally, given the lack of patient diversity in the population this ED serves, future studies should include other clinical sites with different patients population demographics.

## CONCLUSION

In this study, patients in the ED undergoing drug and alcohol use screening were more likely to disclose at-risk alcohol and substance use to a kiosk than an in-person interviewer. In contrast, when patient preferences were surveyed, they stated a preference for an in-person screening over kiosk screening. Comparing findings for substance use screening compared with alcohol screening, we found that alcohol disclosures were also higher via kiosk, although this difference was statistically significant only when comparing participants screened via a single modality, rather than the smaller subset of participants who were screened both via kiosk and in-person. These findings highlight the potential for computerized health screening in the emergency department, especially for health topics that patients may feel uncomfortable disclosing to a clinician. However, our findings also underscore the importance of patient education and interface design to maximize patient comfort with and trust of computerized screening.

## Supplementary Information



## Figures and Tables

**Table 1:** Demographic distribution of participants in each alcohol and drug use screening modality (N=1,207).

Variable	In-person only (n=229) n(%)	Kiosk-only (n=620) n(%)	Dual (n=154) n(%)	p-value
Gender*				
Female	114 (49.8%)	251 (40.5%)	88 (57.1%)	<0.001
Male	115 (50.2%)	369 (59.5%)	66 (42.9%)	-
Race*				
Black	189 (82.5%)	462 (70.9%)	122 (79.2%)	0.02
White	22 (9.6%)	101 (15.5%)	20 (13.0%)	-
Asian	2 (0.9%)	23 (3.5%)	2 (1.3%)	-
Hispanic	5 (2.2%)	22 (3.4%)	1 (0.6%)	-
Other/multiracial	11 (4.8%)	44 (6.7%)	9 (5.8%)	-
Age				
Mean (SD)	35.85 (10.82)	39.31 (18.83)	37.67 (16.13)	0.02
Education*				
<9^th^ grade	10 (4.4%)	26 (4.4%)	2 (1.3%)	<0.0001
Some high school	48 (21.1%)	70 (11.8%)	12 (7.9%)	-
High school	78 (34.2%)	164 (27.6%)	56 (36.8%)	-
Some college	58 (25.4%)	166 (27.9%)	44 (28.9%)	-
College	34 (14.9%)	168 (28.3%)	38 (25.0%)	-
Marital status*				
Single	143 (63.8%)	356 (64.0%)	60 (65.2%)	<0.0001
Separated	24 (10.7%)	30 (5.4%)	7 (7.6%)	-
Divorced	16 (7.1%)	62 (11.2%)	6 (6.5%)	-
Widowed	10 (4.5%)	59 (10.6%)	1 (1.1%)	-
Married	31 (13.8%)	49 (8.8%)	18 (19.6%)	-
Employment				
In school	9 (4.1%)	-	5 (3.2%)	0.52
Employed part-time	38 (17.4%)	-	22 (14.2%)	-
Employed full-time	59 (26.9%)	-	53 (34.2%)	-
Unemployed	93 (42.5%)	-	51 (32.9%)	-
On disability	20 (9.1%)	-	12 (7.7%)	-
Other	10 (4.4%)	-	7 (7.7%)	-

* Statistically significant (p<0.05). Chi-square test of general association and analysis of variance (ANOVA) were used to compare proportions and means, respectively.

**Table 2:** Participant preferences for screening modality (N=383).

Variable	Computer, n(%)	Interviewer, n(%)	No preference, n(%)	Combined, n(%)
Privacy	131 (34.4%)	166 (43.6%)	84 (22.1%)	-
Honesty	135 (35.3%)	186 (48.7%)	61 (16.0%)	-
Comfort	46 (12.1%)	214 (56.2%)	121 (31.8%)	-
Duration	67 (17.8%)	171 (45.4%)	49 (13.0%)	90 (23.9%)

**Table 3:** Capture of total participants and participants at risk for drug/alcohol disorders by screening modality and time of day.

Variable	In-person only	Kiosk-only	Dual
Total			
Business hours	224/227 (98.7%)	358/824 (43.4%)	152/154 (98.7%)
Off-hours	3/227 (1.3%)	466/824 (56.6%)	2/154 (1.3%)
At-risk			
Business hours	16/17 (94.1%)	38/82 (46.3%)	16/17 (94.1%)
Off-hours	1/17 (5.9%)	44/82 (53.7%)	1/17 (5.9%)

* Business hours were categorized as Monday through Friday, 9am–5pm. Off-hours were defined as any other time of day or week.
