# Supplementary material for: Kiosk versus In-person Screening for Alcohol and Drug Use in the Emergency Department: Patient Preferences and Disclosure
Source: West J Emerg Med. 2015 Mar 10;16(2):220–8. doi: 10.5811/westjem.2015.1.24121 (PMC4380369; doi:10.5811/westjem.2015.1.24121)
Supplement: Supplementary file 1 [file wjem-16-220-s001.pdf]

DRUG USE QUESTIONNAIRE (DAST -10)

NAME: ______________________________ Date:___________________

The following questions concern information about your potential involvement with drugs excluding alcohol and tobacco during the past 12 months. Carefully read each countyment and decide if your answer is “YES” or “NO”. Then, check the appropriate box beside the question.

When the words “drug abuse” are used, they mean the use of prescribed or over-the-counter medications used in excess of the directions and any non-medical use of any drugs. The various classes of drugs may include but are not limited to: cannabis (e.g., marijuana, hash), solvents (e.g., gas, paints etc…), tranquilizers (e.g., Valium), barbiturates, cocaine, and stimulants (e.g., speed), hallucinogens (e.g., LSD) or narcotics (e.g., Heroin). Remember that the questions do not include alcohol or tobacco.

Please answer every question. If you have difficulty with a countyment, then choose the response that is mostly right.

**These questions refer to the past 12 months only.**	**YES**	**NO**
1. Have you used drugs other than those required for medical reasons?.....		
2. Do you abuse more than one drug at a time?..		
3. Are you always able to stop using drugs when you want to?.................		
4. Have you had “blackouts” or “flashbacks” as a result of drug use?........		
5. Do you ever feel bad or guilty about your drug use?..............................		
6. Does your spouse (or parent) ever complain about your involvement with drugs?..		
7. Have you neglected your family because of your use of drugs?.............		
8. Have you engaged in illegal activities in order to obtain drugs?............		
9. Have you ever experienced withdrawal symptoms (felt sick) when you stopped taking drugs?...		
10. Have you had medical problems as a result of your drug use (e.g., memory loss, hepatitis, convulsions, bleeding etc…)?.................		
*** DAST Score**………………………………		

** See scoring instructions for correct scoring procedu*

DRUG USE QUESTIONNAIRE (DAST -10)

Administration & Interpretation

Instructions

The DAST-10 is a 10-item, yes/no, self-report instrument that has been shortened from the 28-item DAST and should take less than 8 minutes to complete. The DAST-10 was designed to provide a brief instrument for clinical screening and treatment evaluation and can be used with adults and older youth. It is **strongly recommended** that the SMAST be used along with the DAST-10 unless there is a clear indication that the client uses NO ALCOHOL at all. The answer options for each item are "YES" or "NO". The DAST-10 is a self-administered screening instrument.

Scoring and Interpretation – For the DAST-10, score 1 point for each question answered, "YES", except for question (3) for which a "NO" answer receives 1 point and (0) for a "YES". Add up the points and interpretations are as followed:

DAST-10 Score	Degree of Problem Related to Drug Abuse	Suggested Action
0	No problems reported	None at this time.
1 – 2	Low Level	Monitor, reassess at a later date.
3 – 5	Moderate Level	Further investigation is required.
6 – 8	Substantial Level	Assessment required.
9 – 10	Severe Level	Assessment required.
